# Bone biopsy in nephrology practice

**DOI:** 10.1590/2175-8239-JBN-2017-0012

**Published:** 2018-11-29

**Authors:** Fellype de Carvalho Barreto, Cleber Rafael Vieira da Costa, Luciene Machado dos Reis, Melani Ribeiro Custódio

**Affiliations:** 1 Universidade Federal do Paraná, Divisão de Nefrologia, Curitiba, PR, Brasil.; 2 Fundação Pro-Renal, Curitiba, PR, Brasil.; 3 Universidade de São Paulo, Faculdade de Medicina, Hospital das Clinicas, Laboratório de Fisiopatologia Renal São Paulo, SP, Brasil.

**Keywords:** Biopsy, Bone and Bones, Histology, Bone Histomorphometry, Chronic Kidney Disease-Mineral and Bone Disorder, Renal Insufficiency, Chronic, Biópsia, Osso e Ossos, Histologia, Histomorfometria Óssea, Distúrbio Mineral e Ósseo da Doença Renal Crônica, Insuficiência Renal Crônica

## Abstract

Renal osteodystrophy (ROD), a group of metabolic bone diseases secondary to chronic kidney disease (CKD), still represents a great challenge to nephrologists. Its management is tailored by the type of bone lesion - of high or low turnover - that cannot be accurately predicted by serum biomarkers of bone remodeling available in daily clinical practice, mainly parathyroid hormone (PTH) and alkaline phosphatase (AP). In view of this limitation, bone biopsy followed by bone quantitative histomorphometry, the gold-standard method for the diagnosis of ROD, is still considered of paramount importance. Bone biopsy has also been recommended for evaluation of osteoporosis in the CKD setting to help physicians choose the best anti-osteoporotic drug. Importantly, bone biopsy is the sole diagnostic method capable of providing dynamic information on bone metabolism. Trabecular and cortical bones may be analyzed separately by evaluating their structural and dynamic parameters, thickness and porosity, respectively. Deposition of metals, such as aluminum and iron, on bone may also be detected. Despite of these unique characteristics, the interest on bone biopsy has declined over the last years and there are currently few centers around the world specialized on bone histomorphometry. In this review, we will discuss the bone biopsy technique, its indications, and the main information it can provide. The interest on bone biopsy should be renewed and nephrologists should be capacitated to perform it as part of their training during medical residency.

## Resumo

A osteodistrofia renal (OR), um grupo de doenças ósseas metabólicas secundárias à doença renal crônica (DRC), ainda representa um grande desafio para os nefrologistas. Seu manejo é individualizado de acordo com o tipo de lesão óssea - de alto ou baixo remodelamento - cujo diagnóstico não pode ser prevista com precisão pelos biomarcadores séricos de remodelação óssea disponíveis na prática clínica diária, principalmente o paratormônio (PTH) e a fosfatase alcalina (FA). Em vista dessa limitação, biópsia óssea seguida de histomorfometria óssea quantitativa, método padrão-ouro para o diagnóstico de OR, ainda é considerado um procedimento de grande importância. A biópsia óssea também é recomendada na avaliação da osteoporose em indivíduos com DRC, a fim de auxiliar na escolha do melhor medicamento anti-osteoporótico. É importante observar que a biópsia óssea é o único método diagnóstico capaz de proporcionar informações dinâmicas sobre o metabolismo ósseo. Os ossos trabecular e cortical podem ser analisados separadamente por meio da avaliação de seus parâmetros estruturais e dinâmicos, espessura e porosidade, respectivamente. A deposição óssea de metais como alumínio e ferro também pode ser detectada. Apesar de suas características singulares, o interesse pela biópsia óssea diminuiu nos últimos anos. Poucos centros em todo o mundo são especializados em histomorfometria óssea. A presente revisão discute a técnica de biópsia óssea, suas indicações e as principais informações que ela pode oferecer. O interesse pela biópsia óssea deve ser renovado e os nefrologistas devem ser capacitados a realizá-la durante o período de residência médica.

## Introduction

Abnormalities in circulating parameters of mineral and bone metabolism may appear early in the course of chronic kidney disease and they have been associated to increased mortality and morbidity, and decreased quality of life.^1^^,^ 2 In 2006, the term chronic kidney disease-mineral and bone disorders (CKD-MBD) was implemented by the Kidney Disease Improving Global Outcomes (KDIGO) working group^3^ to refer to the systemic disorder of mineral and bone metabolism due to CKD; it is manifested by either one or a combination of: 1) abnormalities of calcium (Ca), phosphorous (P), PTH and vitamin D metabolism; 2) abnormalities of bone turnover, mineralization, volume (TMV), linear growth or strength; and 3) vascular or other soft tissue calcification. There are many studies showing that these disorders are related to adverse clinical outcomes, in particular cardiovascular disease, fractures, and mortality, reaffirming the importance of this systemic pathological process.^4^^-^ 7

In 2009, KDIGO published clinical guidelines for the diagnosis, prevention, and treatment of CKD-MBD. Renal osteodystrophy (ROD), a group of metabolic bone diseases that occurs through the evolution of CKD, is part of the manifestations of CKD-MBD. The term 'renal osteodystrophy' has been limited to the histologic analysis of bone lesions, requiring the use of bone biopsy.^8^^,^ 9

**Figure 1:**
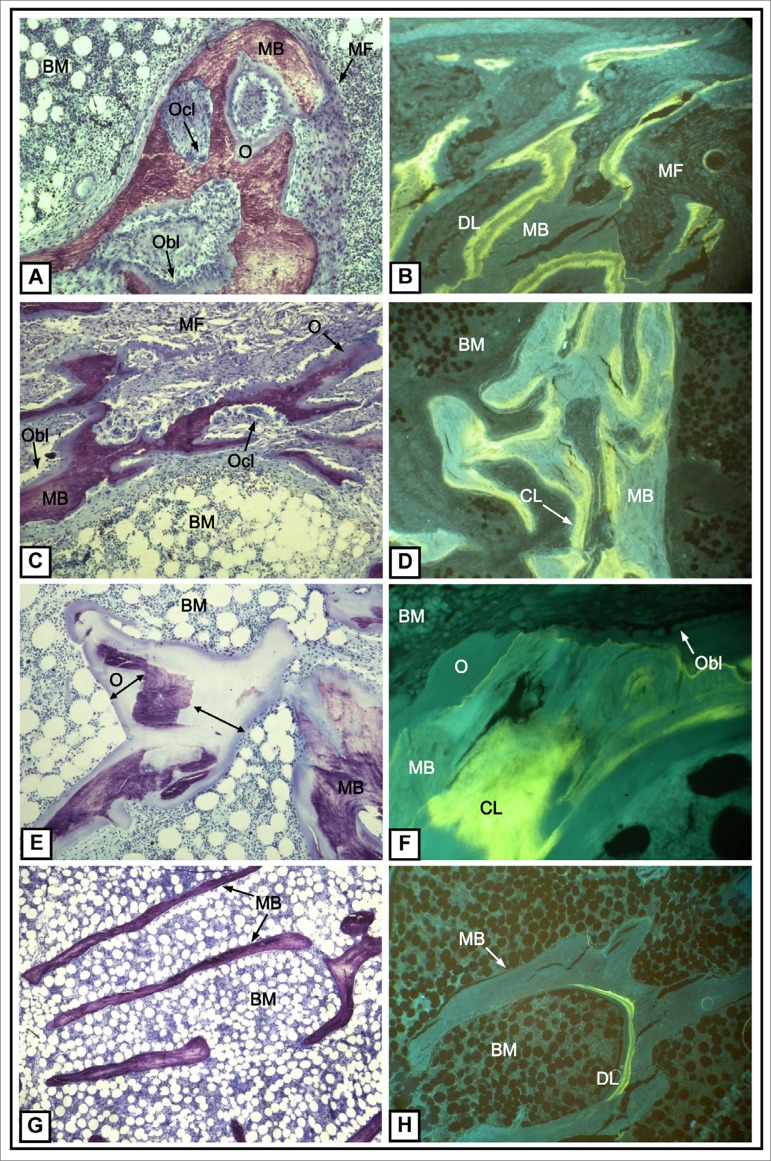
Representative photomicrographs of under-calcified bone showing different types of renal osteodystrophy. MB: mineralized bone; BM: bone marrow. A) Histological characteristics of osteitis fibrosa, showing an increased bone formation represented by osteoid surface (O), osteoblast number (Obl), resorption, and osteoclast number (Ocl). There is an extensive area of marrow fibrosis (MF). Toluidine Blue (x100). B) Fluorescent double-labels (DL) observed in osteitis fibrosa. Unstained bone section under ultraviolet (UV) light (x125). C) Histological characteristics observed in mixed uremic osteodystrophy, represented by an increased bone formation, showing osteoid surface (O), osteoblast number (Obl), resorption and osteoclast number (Ocl), and an extensive area covered by marrow fibrosis (MF). Toluidine blue (x100). D) The major difference between mixed uremic osteodystrophy and osteitis fibrosa is the impaired mineralization observed in this type of disease, as a consequence of the increased confluent labels not observed in osteitis fibrosa. Unstained bone section under UV light (x125). E) Histological characteristics of Osteomalacia, showing a dramatic increase of bone formation represented by an extensive osteoid surface (O) and thickness (arrow). Toluidine blue (x100). F) Confluent fluorescent labels (CL) observed in osteomalacia. Unstained bone section under UV light (x250). G) Histological characteristics observed in adynamic bone disease, showing decreased bone formation and resorption and no marrow fibrosis (MF). Toluidine blue (x40). H) Fluorescent labels in adynamic bone disease can be scarce, representing a decreased mineralization as observed in this unstained bone section under UV light (x50).

Bone biopsy is considered the gold-standard method for histological classification of ROD.^3^^,^ 8 ^-^ 10 It is performed in the iliac crest, after double tetracycline labeling, allowing that the different histological alterations of bone tissue that make up the ROD spectrum can be diagnosed and classified by quantitative histomorphometry. This analysis consists on the evaluation of structural and dynamic parameters, such as trabecular bone volume, bone cell numbers, bone formation, and mineralization. ROD may be classified according to TMV criteria into one of the following patterns: (i) low-turnover bone disease, which includes adynamic bone disease (ABD) and osteomalacia (OM); or (ii) high-turnover bone disease, which encompasses bone disease related to secondary hyperparathyroidism (SHPT), or *osteitis fibrosa*, and mixed uremic osteodystrophy (MUO).^3^^,^ 11 ^,^ 12 Figure 1 shows the different types of ROD and their characteristics. High-turnover bone disease are exemplified in figures 1A, 1B, 1C, and 1D, and low-turnover bone disease in figures 1E, 1F, 1G, and 1H.

Another important analysis is the thickness and porosity of cortical bone. About 80% of all fractures occurs at non-vertebral sites, at regions comprising large amounts of cortical bone. In the past, trabecular bone and vertebral fractures had been the hallmarks of osteoporotic fractures, but recently the role of cortical bone in bone fragility has gained a growing interest.^5^^,^ 13 In the general population, cortical lesions have been consistently associated with increased fracture risk, as is observed in post-menopausal women. Bjornerem *et al* 14 in a high-resolution peripheral CT study, showed that cortical porosity and thickness are the main factors determining the bone fragility that underlies non-vertebral fracture risk.

CKD is associated with higher risk of fractures, being 4x greater in these patients than in the general population, increasing mortality significantly.^15^^-^ 17 One possible explanation is that in CKD there is a decrease in bone mass with impaired bone strength and a high risk of fall, predisposing to fractures.^18^ Taking these data into account, information obtained through quantitative histomorphometric analysis of bone tissue may be help us to understand the mechanisms behind the assoication of bone abnormalities and clinical outcomes, which may be crucial to improve the manangement of bone disease.^9^^,^ 19 Table 1 shows characteristics of ROD according to histomorphometric parameters. Osteoporosis might be present in any type of bone disease, with the exception of osteomalacia.

**Table 1:** Characteristics of renal osteodystrophy according to histomorphometric parameters

Histomorphometric parameters	Osteitis Fibrosa	Mixed bone disease	Adynamic bone disease	Osteomalacia
Structural				
BV/TV (%)	Normal/low	Normal/low	Normal/low	Normal/increased
Tb.Sp (µm)	Normal/increased	Normal/increased	Normal/increased	Normal/decreased
Tb.Th (µm)	Normal/decreased	Normal/decreased	Normal/decreased	increased
Tb.N (/mm)	Normal/decreased	Normal/decreased	Normal/decreased	Normal/increased
formation				
OV/BV (%)	increased	increased	Normal	increased
OS/BS (%)	Normal/increased	Normal/increased	Normal	increased
O.Th (µm)	Normal/increased	Normal/increased	Normal	increased
Ob.S/BS (%)	increased	increased	Normal/decreased	Normal/ decreased
resorption				
ES/BS (%)	Normal/increased	increased	Normal/increased	Normal
Oc.S/BS (%)	increased	increased	Normal/increased	Normal/decreased
mineralization				
MS/BS (%)	Normal	decreased/absent	Normal/decreased	decreased
MAR (µm)	Normal	Normal/increased	Normal/decreased	decreased
BFR/BS (µm^3^/ µm^2^/day)	increased	increased	decreased	decreased
Mlt (days)	Normal/increased	increased	normal/increased	increased
Bone and marrow fibrosis				
Fb.V/TV (%)	yes*	yes*	yes**/no	no

BV/TV: bone volume; Tb.Sp: trabecular separation; Tb.Th: trabecular thickness; Tb.N: trabecular number; OV/BV: osteoid volume; OS/BS: osteoid surface; O.Th: osteoid thickness; Ob.S/BS: osteoblastic surface; ES/BS: resorption surface; Oc.S/BS: osteoclastic surface; MS/BS: mineralizing surface; MAR: mineral aposition rate; BFR/BS: bone formation rate; Mlt: mineralization lag time; Fb.V/TV: fibrosis volume

* higher than 0.5% and

** lower than 0.5%;

Bone biopsy is an invasive and costly procedure that requires specialized centers for histomorphometric analysis. Therefore, it has not been recommended as part of the routine evaluation of CKD-MBD.^9^^,^ 13 The biomarkers of bone remodeling PTH and AP, both total and the fraction, have been considered a helpful tool for predicting ROD type.^8^^,^ 20 Otherwise, some studies have reported that PTH is a poor biomarker of bone disease due to its high biological variability.^20^^,^ 21 In dialysis patients, PTH levels < 100 and > 450 pg/mL have been associated with low- and high-turnover bone disease, respectively, The predictive value of PTH is not considered ideal especially when its level is between 150 - 450 pg/mL, a gray zone where any type of bone histology may occur, including normal bone.^22^ Serum AP is a marker of bone formation. In general, its variation parallels that of PTH, providing additional information for predicting the type of ROD.^23^^,^ 24 In some circumstances, the values of these markers may be divergent, such as high PTH and low total AP levels, which suggests the presence of SHPT associated with a low-turnover bone disease. This apparent paradox can be secondary to aluminum intoxication or over suppression of bone remodeling by active vitamin D therapy. Bone biopsy may be fundamental for clarifying this blurred scenario.^25^

It is worth mentioning that bone biopsy may also provide a complementary view of bone tissue alterations, such as the detection of aluminum and/or iron intoxication and the presence of low bone volume, a finding suggestive of osteoporosis.^26^^,^ 27 Bone biopsy can also provide bone tissue sample for immunohistochemical analysis, molecular studies, data on efficacy and safety of different drugs, among other advantages;^28^^-^ 31 its main indications are listed in Table 2.

**Table 2:** Indications of bone biopsy in CKD patients

1.	Persistent bone pain;
2.	Unexplained hypercalcemia and/or hypophosphatemia;
3.	Fragility fracture;
4.	Discrepancy between serum biomarkers and clinical presentation;
5.	Suspicion of aluminum and/or iron intoxication;
6.	Before using anti-osteoporotic drugs, such as bisphosphonates and denosumab.

Finally, in view of the limitations of the serum biomarkers, other diagnostic methods have been used for the analysis of bone diseases.^32^ Dual energy X-ray absorptiometry (DXA),^33^ quantitative computerized tomography (QCT),^34^ high resolution-peripheral QCT (HR-pQCT)^35^ and micro magnetic resonance imaging (MRI)^36^ are imaging methods that quantify bone mass and structural aspects of bone quality. Importantly, they do not measure neither and mineralization nor determine ROD type. Thus, currently the non-invasive assessment of bone turnover and mineralization is still suboptimal.^20^^,^ 37 Table 3 depicts the advantages and limitations of different methods of bone tissue evaluation.

**Table 3:** Advantages and limitations of different methods for the evaluation of bone tissue

	Advantages	Limitations
Serum biomarkers of bone remodelling	Low cost Availability Non-invasive Provide guidance for the treatment of CKD-MBD in the daily clinical practice	Kidney function may interfere on levels Low to moderate accuracy to predict the type of ROD Lack of information on bone density
DXA	Non-invasive Low cost Availability Prediction of bone fracture	Lack of differentiation between cortical and trabecular bone Lack of information on bone mineralization, turnover and microarchitecture
HR-qCT	Non-invasive High definition image Assesses separately cortical and trabecular bone Assesses bone density and microarchitecture. Estimation of bone strength	High cost, radiation exposure, low availability Limited to peripheral sites Lack of information on bone mineralization and turnover Lack of standardization Few studies in CKD patients
MRI	Non-invasive, no radiation Assesses separately cortical and trabecular bone Can produce 3D images Shows other aspects of bone physiology not assessed by other techniques, such as marrow fat content, perfusion and molecular diffusion	High cost Low availability Lack of information on bone mineralization and turnover Few studies in CKD patients
Bone biopsy + histomorphometry	High specificity and sensibility to diagnose bone diseases, including ROD Assesses separately cortical and trabecular bone Information on bone mineralization and turnover	Invasive, painful, cost Few specialized centres on this technique Does not evaluate bone density No data on fracture risk prediction

Abbreviations: DXA: dual X-ray absorptiometry; HR-qCT, high resolution – quantitative peripheral computerized tomography; MRI, magnetic resonance image.

The update of KDIGO guidelines for CKD-MBD,^8^ in addition to reinforcing all classical indications, calls attention to the importance of bone biopsy to guide treatment in CKD 3a-5D patients with low BMD, and high risk of osteoporosis.

According to a recent survey conducted among European nephrologists, bone biopsies are performed rather exceptionally, both for clinical and research purposes. Many difficulties were identified, especially the costly histopathological analysis, lack of histopathological expertise, and no established reimbursement in several countries.^10^

In March 2016, the European Renal Osteodystrophy (EU-ROD) initiative was created to revitalize bone biopsy as a clinically useful tool in the diagnosis of CKD-MBD to improve outcomes in CKD patients.^10^ In Brazil, Oliveira RB *et al* created the Brazilian Registry of Bone Biopsy (REBRABO), a national registry that serves as a research platform to expand the knowledge about CKD-MBD.^38^

In the next sections of article, we will overview the protocol of tetracycline labeling, and the technique to perform bone biopsy, its indications and complications. Finally, the main information provided by bone histomorphometric analysis will be discussed.

### Double tetracycline labeling

Patients should take tetracycline previously to the bone biopsy for an adequate evaluation of the type of bone disease. Tetracycline, a macrolide antibiotic, deposits on bone surface allowing the assessment of dynamic parameters of bone formation and mineralization. The tetracycline administration scheme we have been using in our services is the following: first course of tetracycline for 3 days, 500 mg twice a day; a tetracycline-free interval of 10 days; a second course of tetracycline for 3 days, 500 mg twice a day. Bone biopsy must be performed from 3 to 5 days after the end of the second course of tetracycline.^9^^-^ 11

### Bone biopsy material

**Figure 2:**
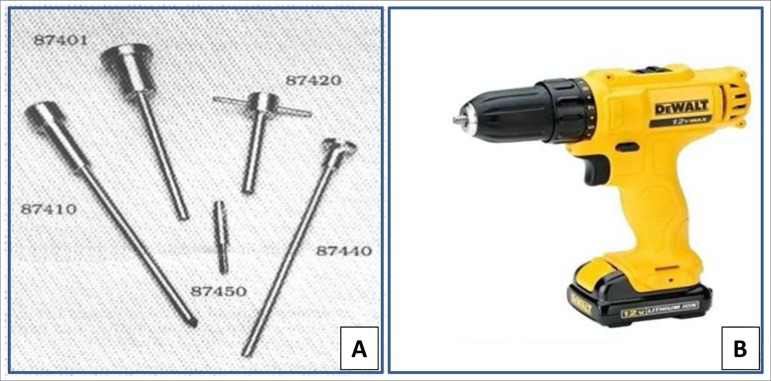
Bone biopsy materials. A-Trephine: Rochester Bone Biopsy Trephine (www.medicalinnovations.com) pointed obturator (number 87410), guide sleeve (number 87420), drive adaptor (number 87450), trephine needle (number 87401), and blunt extractor (number 87440) B-Electric drill: DeWALT®, wireless, 1.2 kg, 12 V Lithium Battery Bivolt Dcd700l

The material required for performing bone biopsy is (*i*) a minor surgery pack containing 1 Kelly thin straight tweezer, 1 rat tooth tweezer, 1 small bowl, 1 medium straight scissor, 1 needle holder, 1 syringe (20 mL); 1 suture thread (3.0); 1 bistoury blade; (*ii*) 1 set of trephine needle for bone biopsy; and (*iii*) 1 electric drill. Figure 2 illustrates these materials.

### Transiliac bone biopsy procedure

Transiliac bone biopsy can be performed in an ambulatory surgical center in most cases. Mild sedation with midazolam, administered by intravenous or intramuscular route, should be used. Performing bone biopsy in obese patients and in children may require in-hospital surgical center and the presence of anesthesiologist.

The first step of bone biopsy procedure is to define the correct site on the iliac crest. The superior iliac crest should be located and imaginary lines drawn 2 cm below and 2 cm inwards toward the contralateral shoulder, forming a 90º angle with the iliac bone.

Asepsis should be made before starting the procedure with, for example, alcoholic chlorhexidine. For local anesthesia, a button on the skin of around 2 cm should be made in the incision site. The anesthetic should be injected along the biopsy path and into the periosteum of the external cortical. The internal cortical bone may also be anesthetized by entering with a needle 2 cm below and posterior to the iliac crest. In the previously defined point, a 2 cm incision should be made. Thereafter, the muscle plans should be gently divulged to avoid bleeding, using a straight Kelly to make a tunnel from the skin until the periosteum. Once the tunnel is made, the Rochester Bone Biopsy kit that includes a pointed obturator (number 87410), a guide sleeve (number 87420), a drive adaptor (number 87450), a Trephine cutter (number 87401) and a blunt extractor (number 87440) (Figure 2) will be used for the following steps:

1. The pointed obturator should be inserted through the incision until reaching the surface of the ilium to further localize the biopsy site.
2. The pointed obturator should be removed and connected to the guide sleeve. The assembly should be reintroduced into the tunnel until the previously determined site of biopsy. The guide sleeve should be held firmly while the pointed obturator is withdrawn.
3. The Trephine needle should be connected to the drive adaptor and coupled to the drill, being firmly fastened to the drill.
4. The assembly should be inserted into the guide sleeve, which acts as a catch lock. the Trephine must not exceed the guide sleeve more than 3 cm to assure that it will not excessively overpass the ilium.
5. The drill should be driven forward rotating in the clockwise direction. The Trephine needle should pass through the external cortical, trabecular, and internal cortical bone, to obtain an adequate bone sample. Thereafter, the direction of the drill rotation should be set to counterclockwise and the drill driven backwards.
6. The bone fragment should be removed from the trephine needle using the blunt extractor.
7. The incision wound should be compressed for 5 minutes to stop bleeding.
8. The incision should be cleaned and sutured using Donati technique.
9. A compressive dressing should be made to prevent bleeding.

The bone fragment size should be variable, depending on factors such as gender and patient size. It is considered adequate when the fragment is composed of two cortical (internal and external) bones at the extremities and trabecular bone in the middle. Importantly, the bone specimen should not be fractured during the procedure. The sample should be stored in a vial containing 70% alcohol and protected from light exposure. Formalin should not be used because it may damage the bone tissue.

### Complications of bone biopsy

The transiliac bone biopsy has been proved to be safe and associated with minor complications, which incidence is of 0.63%.^39^^,^ 40 The most common complications are bleeding, hematoma, infection, superficial nerve injury, and pain. Routine prophylactic antibiotic administration is not recommended. Antibiotics should be reserved for the cases of excessive manipulation or accidental contamination. The main recommendations for patient after bone biopsy are listed in Table 4.

**Table 4:** Patient recommendations after bone biopsy procedure

•	The patient must take a day off
•	The patient should not perform heavy tasks for the following 3 days.
•	The dressing should be removed 24 hours after the procedure.
•	The surgical wound can be washed with soap and water.
•	A new dressing or a Band-Aid may be used.
•	Painkillers, such as dipyrone or paracetamol, for alleviating pain.
•	Heparin should be avoided during the hemodialysis session following the bone biopsy.
•	The stitches should be removed 7 - 10 days after the procedure.

### Histomorphometric analysis

The bone biopsy result is reported according to the TMV classification. Briefly, it indicates if bone turnover (T) and volume (V) are low, normal, or increased, and if the mineralization (M) is normal or abnormal. The high-turnover bone diseases comprise SHPT-related bone disease (Figure 1A and 1B) or MUO (Figure 1C). Both are characterized by an increase in the number and activity of bone cells (osteoblasts and osteoclasts) and, consequently, higher bone formation and resorption. (Table 1) The presence of bone marrow fibrosis is a common finding in high-turnover state (Table 1). The main difference between these two patterns of high-turnover bone disease is the presence of abnormal mineralization in MUO (Figure 1 D; Table 1). Bone volume may be decreased in any type of ROD (excepted osteomalacia), indicating a predominance of bone resorption over bone formation (Table 1) and greater brittleness of bone tissue. Low-bone turnover disease is characterized by low cellularity (reduced number of osteoclasts and osteoclasts). Low bone formation and mineralization (Figure 1G and 1H; Table 1) are the main finding of ABD while a greater impairment of bone mineralization, leading to the accumulation of the osteoid matrix, is the main finding of osteomalacia (Figure 1F; Table1). Finally, it should be mentioned that despite being commonly performed in trabecular bone, the histomorphometric analysis may also be used to evaluate the cortical bone. Cortical thickness and porosity may be assessed by this method. Lower thickness and greater porosity of the cortical bone indicate greater fragility and, therefore, greater risk of fracture. The presence of aluminum deposits can be evaluated by Solochrome Azurine and iron by Perls' Prussian Blue staining.

### Centers for bone histomorphometry analysis in Brazil

Currently, there are four centers specialized on bone histomorphometry in Brazil, three located in São Paulo and one in Paraná. They are:

1. Laboratório de Fisiopatologia Renal - Lim 16 - Faculdade de Medicina da Universidade de São Paulo (FMUSP).
2. Laboratório de Osteodistrofia Renal - Disciplina de Nefrologia - Universidade Federal de São Paulo (UNIFESP).
3. Laboratório PRO, Fundação Pró-Renal Brasil - Curitiba.
4. Laboratório para Estudo do Distúrbio Mineral e Ósseo em Nefrologia (LEMON) - disciplina de Nefrologia - Universidade de Campinas (UNICAMP).

## Conclusion

Bone biopsy is an important procedure that provides helpful information on bone microarchitecture, turnover and, ultimately, on the type of ROD. This information may be critical for choosing the most appropriate therapy for CKD-MBD and other findings as osteoporosis, aluminum and/or iron intoxication. Nephrologists should be capacitated to perform bone biopsy as part of their training during medical residency.
